# Molecular Characterization and Clinical Characteristics of m5C-Based RNA Methylation in Spinal Cord Injury: Validated by qPCR

**DOI:** 10.1155/2022/5433860

**Published:** 2022-12-20

**Authors:** Liang Cao, Wen Jun Pi, Qiang Zhang, Qing Li

**Affiliations:** ^1^School of Clinical Medicine, Guizhou Medical University, Guiyang 550025, China; ^2^Department of Orthopedics Traumatic, The Affiliated Hospital of Guizhou Medical University, Guiyang, China

## Abstract

Aberrant patterns of 5-methylcytosine (m5C)-based ribonucleic acid (RNA) methylation have critical roles in various human diseases, but their importance in spinal cord injury (SCI) is largely unknown. We explore the expression patterns and potential roles of m5C-based regulators of RNA modification after SCI. We analyzed 16 m5C-based regulators of RNA modification in tissues with SCI and normal rats from the Gene Expression Omnibus database. We constructed a “gene signature” of m5C-based regulators of RNA modification to predict the prognosis of SCI using least absolute shrinkage and selection operator regression and random-forest strategy. We found that the m5C-related genes, deoxyribonucleic acid (DNA) methyltransferase1 (*Dnmt1*), methyl-CpG binding domain protein 2 (*Mbd2*), ubiquitin-like with PHD and ring finger domains 1 (*Uhrf1*), uracil-*N*-glycosylase (*Ung*), and zinc finger and BTB(brica-brac, tramtrack, and broad) domain containing 38 (*Zbtb38*) had high expression, and zinc finger and BTB domain containing 4 (*Zbtb4*) had low expression in SCI. Analysis of the correlation between the gene sets of m5C-based regulators of RNA modification and immune-cell infiltration and immune response revealed Dnmt1, DNA methyltransferases 3A (Dnmt3a), Mbd2, and Ung to be positive regulators of the immune microenvironment, and Zbtb4 may negatively regulate the immune environment. Then, two molecular subtypes were identified based on 16 m5C-regulated genes. Functional-enrichment analysis of differentially expressed genes between different patterns of m5C-based modification was undertaken. Through the creation of a protein–protein interaction network, we screened 11 hub genes. We demonstrated their importance between SCI group and sham group using real-time reverse transcription-quantitative polymerase chain reaction in rat model. Expression of hub genes did not correlate with mitophagy but was positively correlated with endoplasmic reticulum stress (ERS), which suggested that there may be differences in ERS between different patterns of m5C-based modification. This present study explored and discovered the close link between m5C regulators-related genes and SCI. We also hope our findings may contribute to further mechanistic and therapeutic research on the role of key m5C regulators after SCI.

## 1. Introduction

Spinal cord injury (SCI) is severe damage to the central nervous system. SCI leads to the loss of sensory and motor capabilities and seriously affects physical and mental health [1, 2]. In terms of severity, SCI can be divided into “traumatic” and “non-traumatic” types [3]. Traumatic SCI is attributed to direct and immediate mechanical damage to the spinal cord, and its causes mainly include motor vehicle accidents (MVA), violence, falls, and sports-related injuries [4]. Non-traumatic SCI is caused by insufficient blood flow, spinal tumors, or osteoarthritis [5–7]. SCI pathophysiology includes ischemia, oxidative stress, inflammation, apoptosis, and motor dysfunction [1]. However, SCI is considered to be an incurable disease [8]. Therefore, understanding the pathophysiology of SCI is essential for developing efficacious treatments.

Ribonucleic acid (RNA) modifications have vital roles in many biological processes (BPs) [9–11]. Moreover, methylation has been identified as a common phenomenon and a key regulator of gene transcription. 5-Methylcytosine (5mC) is a methylated form of the deoxyribonucleic acid (DNA) base cytosine. m5C occurs mainly in the anticodons of transfer-RNAs and ribosomal-RNAs as well as the loop and variable regions and coding sequences for messenger (m)RNAs [12–15]. Studies have demonstrated that mRNA modification, especially m5C methylation, can regulate the nuclear effects of mRNAs, differentiation of neural stem cells, and maintenance of mRNA stability.

RNA modifications also have essential roles in SCI. Studies have demonstrated that methyltransferase-like 14 (Mettl14) suppresses the expression of Ras-related dexamethasone-induced-1 and induces the apoptosis of spinal cord neurons during SCI. Crosstalk between fat mass and obesity-associated protein (FTO) and matrix metallopeptidase 24 (Mmp24) mRNA can facilitate MMP24 translation in the spinal cord and, ultimately, lead to the genesis of neuropathic pain. Ubiquitin-specific protease 18 (USP18) can positively regulate reactive astrogliosis, thereby leading to widespread inflammation and poor functional recovery after SCI. Mettl14 can promote the apoptosis of spinal cord neurons and exacerbate SCI by inhibiting eukaryotic protein translation elongation factor 1*α*2 (EEF1A2) expression. Therefore, RNA modifications in the spinal cord may have important roles in the progression and deterioration of SCI. However, under SCI conditions, the “landscape” and potential role of m5C-based modifications of mRNAs are unclear.

We explored the expression of m5C-based regulators of RNA modifications using the Gene Expression Omnibus (GEO) database. Sixteen m5C-based regulators of RNA modifications were identified from the literature. Using more abundant bioinformatic analysis, including differential expression, analysis of RNA modification in SCI, correlation analysis of RNA modification and immune characteristics, unsupervised clustering analysis, differential and functional enrichment evaluation, protein–protein interaction (PPI), and gene set variation analysis (GSVA), we attempted to construct a complete picture of m5c regulators in SCI. Lastly, we validated the expression of key SCI-related m5C regulators in animal tissues. In summary, this present study will provide valuable direction for further research on the molecular mechanism of epigenetic alterations of SCI development. It would be essential in identifying novel targets for early detection and treatment of SCI.

## 2. Methods

### 2.1. Data Acquisition and Analysis of Differential Expression

The SCI-related datasets GSE20907 [16] and GSE45006 [17] were downloaded from the GEO database (http://www.ncbi.nlm.nih.gov/geo/). Both datasets are from *Rattus norvegicus*. The data platform of GSE20907 is GPL6247 and comprises 24 samples. The data platform of GSE45006 is GPL1355 and contains 24 samples. We included the same treated samples in the two datasets (four controls and four samples after SCI in GSE20907, and four controls and two samples after SCI in GSE45006) for a total of eight controls and six SCI samples. First, the two datasets were normalized separately. Then, the required samples were extracted and combined, and GSVA was used to remove batch effects.

To investigate the alteration of *m5C* expression between SCI tissue and normal tissue, 16 regulatory factors of m5C were identified from the literature [18–20]. These regulatory factors of m5C were: DNA methyltransferase1 (Dnmt1), DNA methyltransferases 3A (Dnmt3a), methyl-CpG binding domain protein 2 (Mbd2), ubiquitin-like with PHD and ring finger domains 1 (Uhrf1), uracil-*N*-glycosylase (Ung), zinc finger and BTB domain containing 38 (Zbtb38), zinc finger and BTB domain containing 4 (Zbtb4), methyl-CpG binding domain protein 1 (Mbd1), methyl-CpG binding domain protein 3 (Mbd3), DNA methyltransferases 3B (Dmt3b), methyl-CpG binding domain protein 4 (Mbd4), methyl CpG binding protein 2 (Mecp2), Nei endonuclease VIII-like1 (Neil1), *N*th like DNA glycosylase 1 (*N*thl1), thymine DNA glycosylase (Tdg), and ubiquitin-like with PHD and ring finger domains 2 (Uhrf2). According to information on sample grouping, we undertook differential analysis using the package “limma” within R (R Institute for Statistical Computing, Vienna, Austria) [21]. The results of differential genes analysis were visualized by the packages “heatmap” and “ggplot2” within R.

### 2.2. Analysis of m5C-Based Regulators of RNA Modification in SCI

The relationship between the 16 m5C-based regulators of RNA modification in SCI samples was evaluated by Spearman's correlation analysis. SCI-related m5C regulators were identified using the random-forest (RF) approach. Least absolute shrinkage and selection operator (LASSO) regression is a widely used machine-learning method for building diagnostic models. “Regularization” is employed to solve overfitting during curve fitting and improve the accuracy of the model. Feature selection and dimensionality reduction were undertaken using LASSO regression [22] and yielded an m5C modifier-dependent SCI classifier. A nomogram was used to analyze and identify the risk factors associated with SCI.

### 2.3. Correlation Analysis of m5C-Based Regulators of RNA Modification and Immune Characteristics

Single-sample gene set enrichment analysis (ssGSEA) [23] is employed to assess the number of specific infiltrating immune cells and the activity of a specific immune response. ssGSEA defines an “enrichment score” to represent absolute enrichment in a gene set in each sample within a given dataset. A list of the genes of infiltrating immune cells was obtained from a study by Shen et al. [24]. A gene set for the immune response was obtained from the ImmPort database (http://www.immport.org/) [25]. This “enrichment fraction” represents the absolute enrichment of immune cells and responses. Spearman's correlation analysis was employed to determine the correlation of m5C-based modulators with the percentage of immune cells and immune reactivity.

### 2.4. Unsupervised Clustering Analysis of Patterns of m5C-Based Modification

According to the gene expression of 16 m5C regulators, we wanted to explore whether there were different modification patterns after SCI. Clustering was undertaken using “ConsensuClusterPlus” within R [26], and the number of clusters and robustness was evaluated. Principal component analysis (PCA) verified the expression patterns of 16 m5C regulators under different modification patterns.

### 2.5. Differential Analysis of Patterns of m5C Modification

We used the “limma” package to analyze the differences between two patterns of m5C modification. The differential analysis results were displayed *via* the R package “pheatmap” to draw heatmaps and ggplot2 to draw volcanic maps. In this study, these genes with |log_2_ fold change| > 1.5 and *p* < 0.05 were considered to be differentially expressed genes between different patterns of m5C-based modification.

### 2.6. Functional Enrichment of Differentially Expressed m5C-Associated Genes

Biological changes can be reflected in biological signaling pathways. The Gene Ontology (GO) database (http://geneontology.org/) was employed for analysis of the functional enrichment of genes concerning BP, molecular function (MF), and cellular component (CC). The Kyoto Encyclopedia of Genes and Genomes (KEGG; http://www.genome.jp/kegg/) is employed to analyze the enrichment of signaling pathways. Using the “clusterProfiler” package [27], functional enrichment and signaling-pathway enrichment were analyzed on the differentially expressed genes related to different patterns of m5C-based modification.

### 2.7. The Construction of PPI Networks and Screening of Hub Genes

The Search Tool for the Retrieval of Interacting Genes/Proteins (STRING) database (https://string-db.org/) [28] was used for searching known and predicted interactions between proteins. These data of STRING were obtained from experimental data, text-mined results from PubMed abstracts, and synthesis of other database data; and predicted results using bioinformatics analysis. We used the STRING database to construct a PPI network for differentially expressed genes related to patterns of m5C-based modification. Subsequently, we imported the PPI network of the STRING database into Cytoscape (https://cytoscape.org/), used the “cytoHubba” plugin to screen 11 hub genes, and analyzed the correlation between these 11 hub genes.

### 2.8. Creation of an SCI Model

The protocol for the animal experiment was approved by the Animal Care and Use Committee of Guizhou Medical University (Guizhou, China). We obtained Sprague Dawley rats (6–8 weeks) from the Experimental Animal Center of Guizhou Medical University (license number: SCXK [Qian] 2018-0001). In this study, all the experimental animals were divided into the SCI and sham groups. First, all rats were anesthetized with 1.25% tribromoethanol. We carefully performed the laminectomy of the T10 vertebra. Then, the spinal cord was crushed with a vessel clamp for 15 seconds. Once the emergence of paralysis of both lower limbs indicated the successful modeling of SCI. We performed the same treatment for the sham group without injuring the rats. After surgery, these rats would be returned to their cages and carried out manual bladder expression twice a day. After 14 days, all rats were sacrificed and used for further experiments.

### 2.9. Gene Set Variation Analysis

It has been reported that mitophagy and endoplasmic reticulum stress (ERS) have essential roles in SCI [29, 30]. Hence, we collected the genes associated with mitophagy and ERS in the “c5.go.v7.4.symbols” gene set. The expression matrix was analyzed by GSVA [31] to calculate the enrichment score of the relevant pathway, and the correlation with the hub gene was calculated.

### 2.10. Real-Time Reverse Transcription-Quantitative Polymerase Chain Reaction

We extracted the total RNA from the rats' spinal cords using TRIzol^®^ Reagent (Tiangen Biotech, Beijing, China). According to manufacturer instructions, we polyadenylated and reverse-transcribed the RNA into complementary DNA using a poly(T) adapter. Real-time polymerase chain reaction (PCR) was performed using a thermal cycler under the following parameters: a 5-minute initial denaturation step at 95°C; 44 cycles at 95°C for 15 seconds; 55°C for 30 seconds; and 72°C for 20 seconds.  Table 1 lists all primer information for mRNA.

### 2.11. Statistical Analysis

The statistical analysis was undertaken using R 4.0.2. The Student's *t*-test estimated the significance of variables in two groups with a normal distribution, and the Mann–Whitney *U*-test analyzed the differences among variables with a non-normal distribution. In this study, *p* < 0.05 (two-sided) was considered significant.

## 3. Results

### 3.1. Expression of m5C-Related Genes in SCI

**Figure 1:**
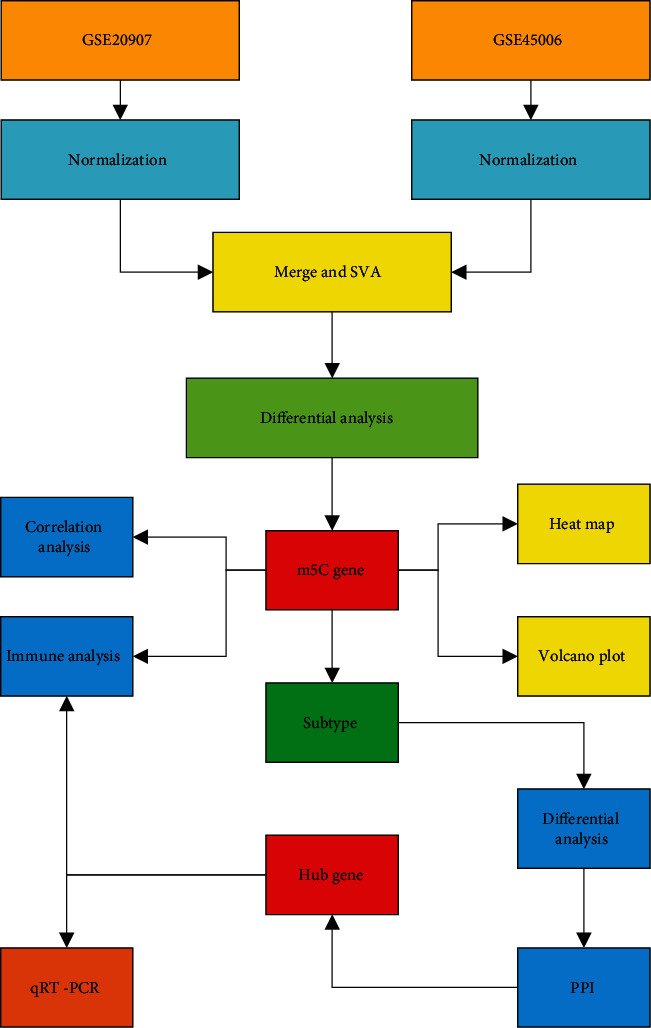
Flowchart of the present study.

**Figure 2:**
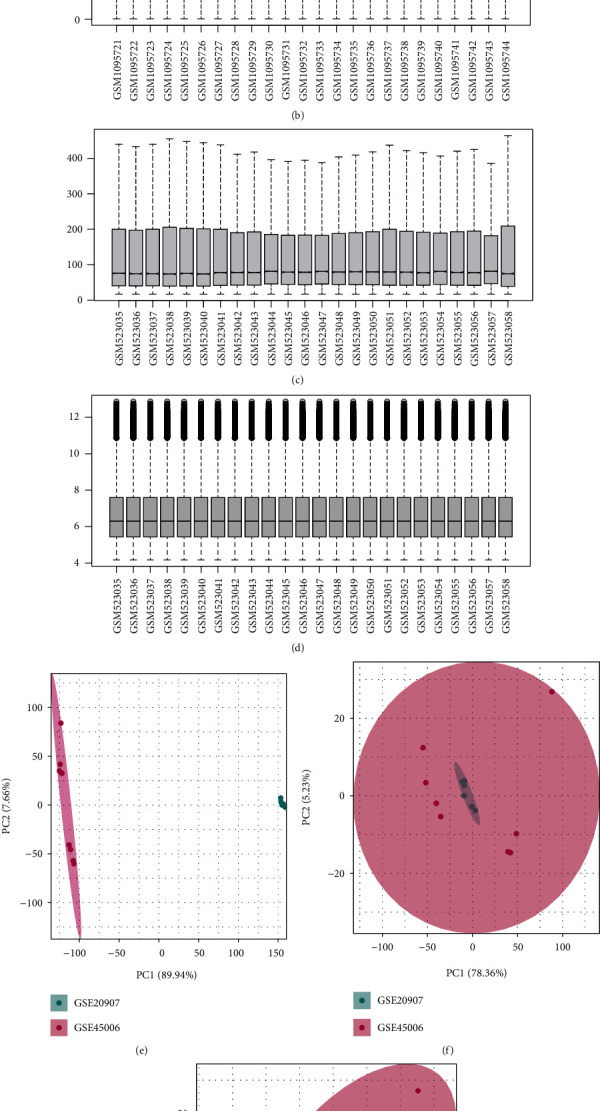
Dataset processing. (a) Boxplot using the raw data of GSE45006. (b) Boxplot after normalization of data of GSE45006. (c) Boxplot using the raw data of GSE20907. (d) Boxplot after normalization of data of GSE20907. (e) Principal component analysis (PCA) plot before GSVA after dataset merging. (f) PCA plot after GSVA after dataset merging. (g) Dataset merging and PCA plots of the disease group and control group after GSVA.

**Figure 3:**
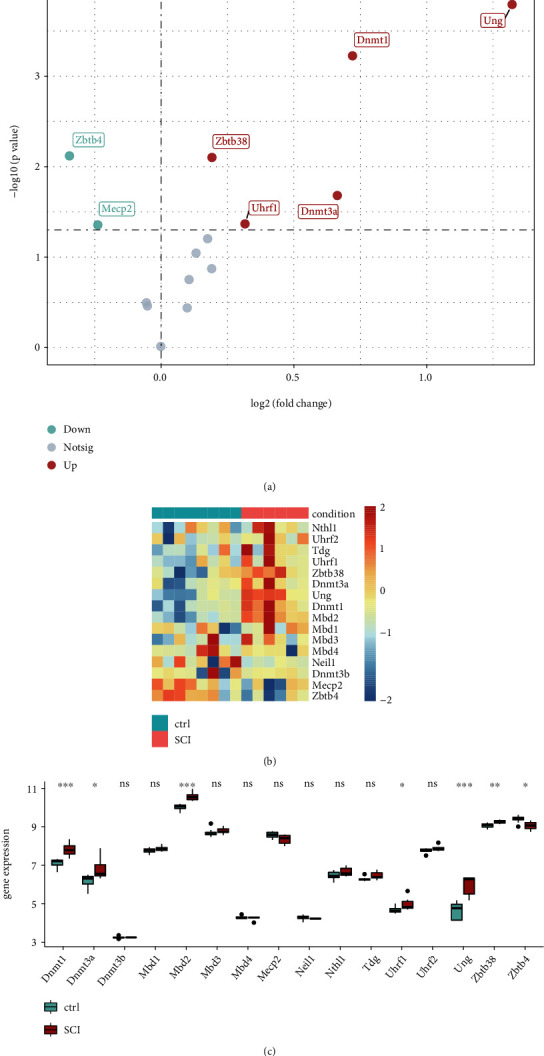
Differences in expression of m5C-associated genes in spinal cord injury using different types of plot. (a) Volcano plot. The abscissa is log2FC and the ordinate is −log10 (*p*-value). (b) Heatmap. (c) Boxplot (^ns^*p* > 0.05, ∗*p* < 0.05, ∗∗*p* < 0.01, and ∗∗∗*p* < 0.001).

Figure 1 is a flowchart of the present study. Histograms showing dataset processing and merging of PCA graphs is shown in Figure 2. We wished to analyze the effect of m5C-related gene expression on SCI tissues compared with that in normal tissues. Hence, we used the “limma” package within R to examine differences in expression of m5C-associated genes in the dataset and drew a volcano plot (Figure 3(a)). We discovered that Zbtb4 and *Mecp2* had low expression and that *Dnmt*1, *Dnmt3a*, *Mbd*2, *Uhrf*1, *Ung*, and *Zbtb38* had the high expression in SCI group. Simultaneously, we drew a heatmap (Figure 3(b)) and a box plot (Figure 3(c)) according to the expression of m5C-related genes: *Dnmt*1, *Mbd2*, *Uhrf*1, *Ung*, and *Zbtb38* had the high expression, and *Zbtb*4 had low expression in SCI group.

### 3.2. Correlation of Expression of m5C-Related Genes in SCI

**Figure 4:**
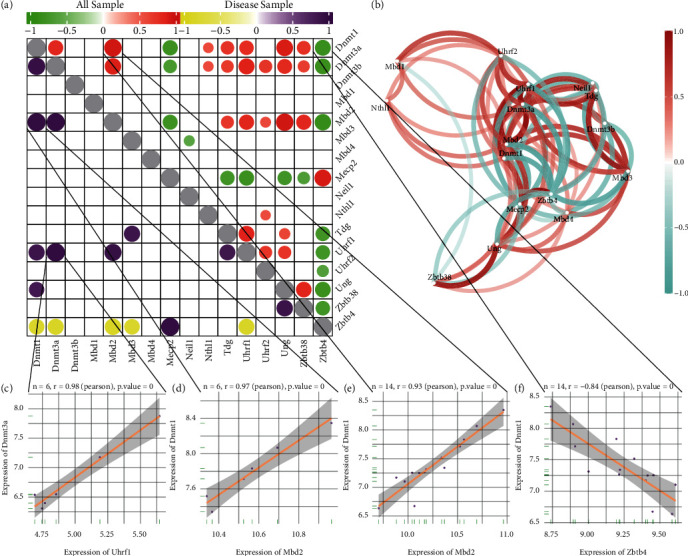
Correlation of expression of 5-methylcytosine (m5C)-related genes in spinal cord injury (SCI). (a) Heatmap of the correlation of expression of 23 m5C-related genes. The upper-right half shows the correlation in all samples. The lower-left half shows the correlation in a SCI sample. The size and color of the nodes represent the size of the correlation, and only nodes with *p* < 0.05 are shown. (b) Network diagram showing correlation of expression of m5C-related genes. (c)–(f) Scatter plots of some m5C-related genes with high correlation in expression.

We undertook correlation analysis of the expression of m5C-related genes, and the results were visualized as a circle plot (Figure 4(a)) and network plot (Figure 4(b)). The upper part of the heatmap is the correlation in all samples, and the lower part is the correlation in the SCI sample. Finally, we revealed the correlation in expression as scatter plots between the most strongly correlated genes (Figures 4(c), 4(d), 4(e), and 4(f)).

### 3.3. Construction of a Prediction Model for m5C-Related Genes in SCI

**Figure 5:**
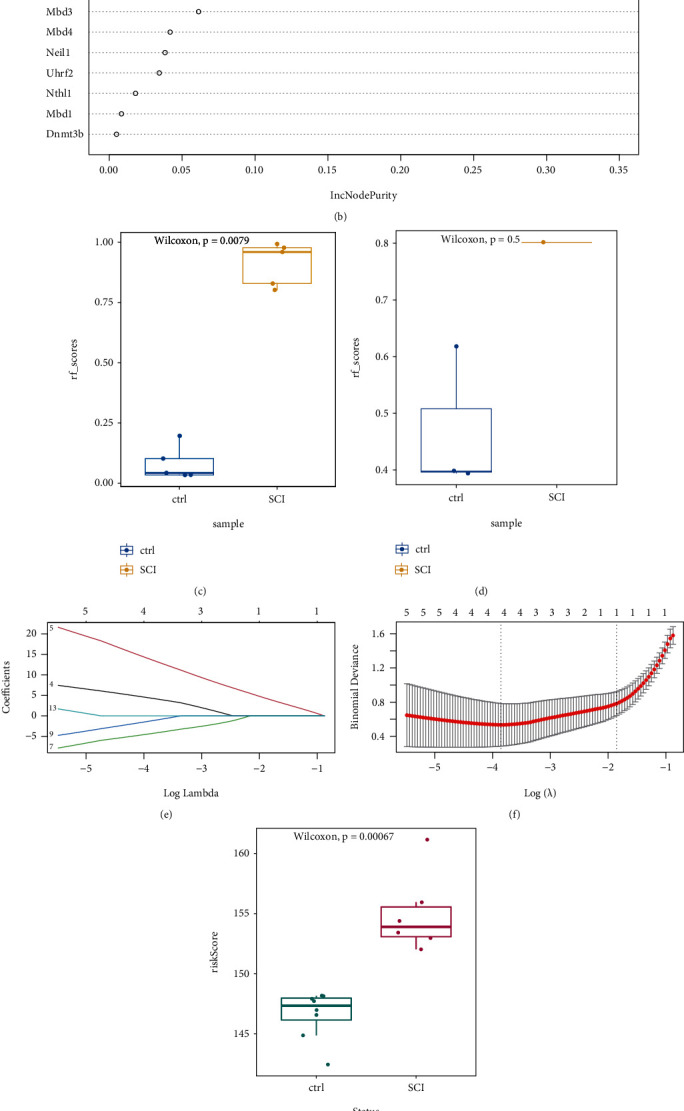
Analysis using the random-forest (RF) method and least absolute shrinkage and selection operator (LASSO) regression. (a) and (b) RF method to model 5-methylcytosine (m5C)-related genes. (c) and (d) Boxplots of ratings for the training set and validation set. (e) and (f) Modeling of m5C-related genes using LASSO regression. (g) Boxplot using LASSO regression. (h) Diagnostic nomogram.

We wished to analyze the discriminatory ability of m5C-related genes in SCI for SCI. First, we undertook analysis using the RF method (Figures 5(a) and 5(b)). Samples were split into a training set (70%) and validation set (30%). Boxplots (Figures 5(c) and 5(d)) showed a significant difference in model scores between the SCI group and healthy group in the training set and validation set, respectively. Results using the RF approach showed that all m5C-associated genes had good predictive ability. Then, we used LASSO regression to screen the feature variables (Figures 5(e) and 5(f)). The obtained prediction model was:

(1) $$\begin{array}{c} \text{Risk score}=\text{Mbd}1*4.291+\text{Mbd}2*13.629+\text{Mbd}4*-4.267+\text{Neil}1*-1.157. \end{array}$$

The boxplot showed a significant difference in risk score between the SCI group and the healthy group (Figure 5(g)). Subsequently, we constructed a nomogram associated with SCI risk (Figure 5(h)).

### 3.4. Interrelationship of m5C-Associated Genes with the Abundance of Immune Cells and Immune Processes after SCI

**Figure 6:**
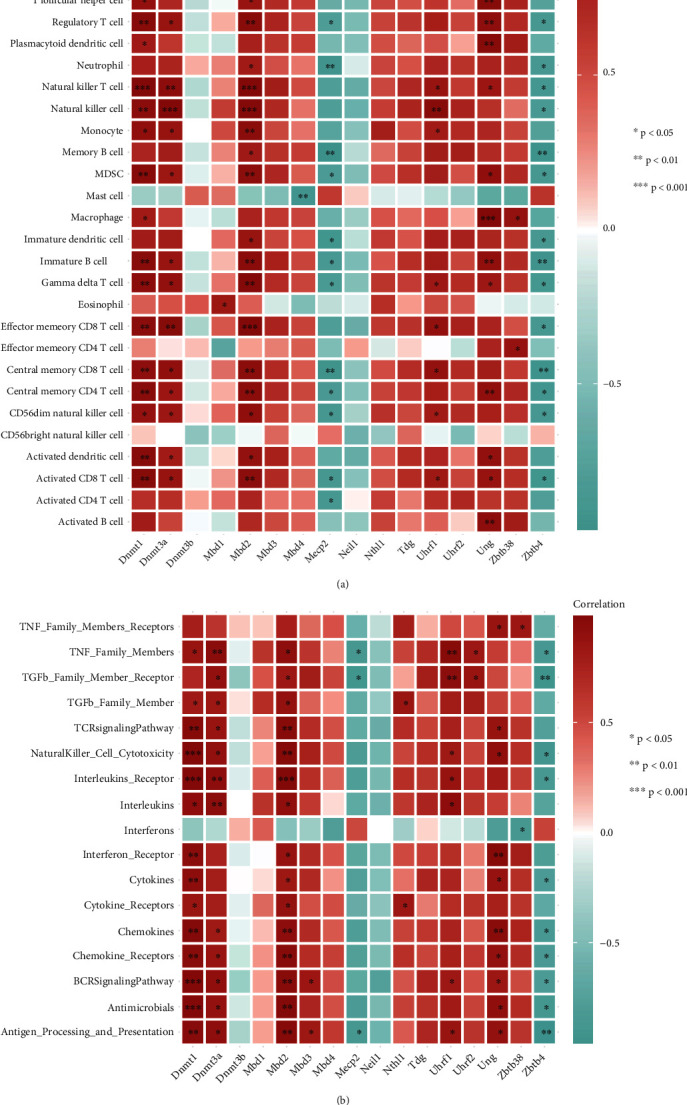
Correlation between the abundance of infiltrating immune cells, immune-response genes, and 5-methylcytosine (m5C)-based regulators of RNA modification. (a) Heatmap showing the correlation between each type of aberrant infiltrating cell in the immune microenvironment and each m5C-based regulator of RNA modification. (b) Dot plot showing the correlation between each genome in the immune process and each m5C-based regulator of RNA modification.

We wished to investigate the correlation between m5C-based regulators of RNA modification and the immune microenvironment. We undertook correlation analysis of m5C-based regulators of RNA modification with the gene sets associated with immune-cell infiltration and the immune response (Figures 6(a) and 6(b)). Results revealed a correlation between the abundance of 23 types of infiltrating cells in the immune microenvironment in SCI specimens and expression of m5C-based regulators of RNA modification. Expression of Dnmt1, Dnmt3a, Mbd2, and Ung was positively correlated with most immune cells, whereas expression of Mecp2 and Zbtb4 was negatively correlated with the abundance of immune cells. Similar to the immune microenvironment during immunization, expression of Dnmt1, Dnmt3a, Mbd2, and Ung was positively correlated with most immune processes, whereas Zbtb4 expression was negatively correlated with immune processes. These data suggested that Dnmt1, Dnmt3a, Mbd2, and Ung were positive regulators of the immune microenvironment, and Zbtb4 could negatively regulate the immune environment.

### 3.5. Pattern of m5C-Based RNA Modification Mediated by 16 Regulatory Factors in SCI

**Figure 7:**
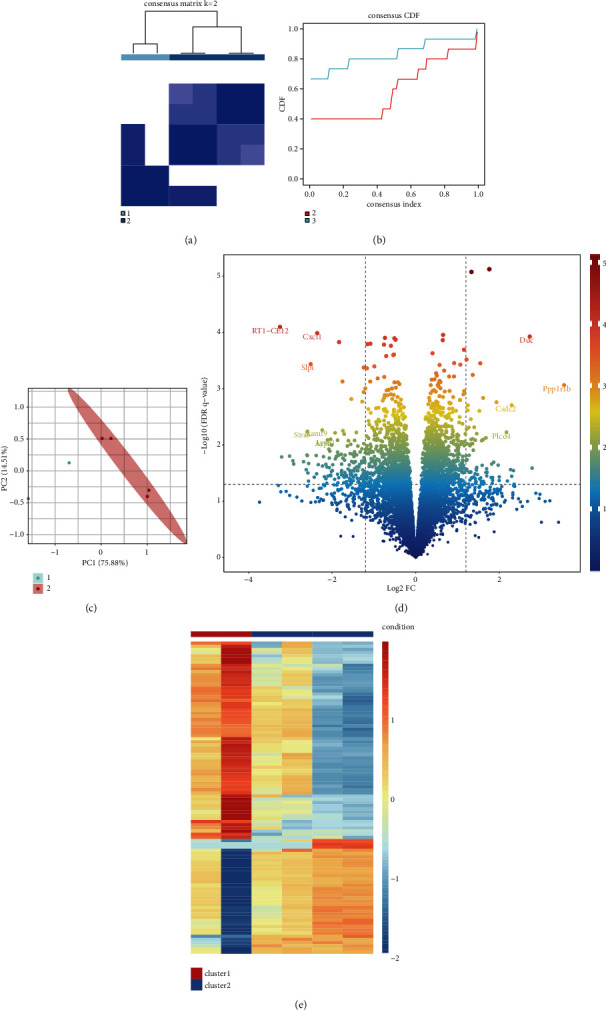
Unsupervised clustering and subtype analysis of 16 5-methylcytosine (m5C)-based regulators of RNA modification. Identification of two distinct patterns of m5C modification in spinal cord injury (SCI). (a) Heatmap showing the co-occurrence ratio matrix of SCI samples if *K* = 2. (b) Relative change in area under the CDF(cumulative distribution function) curve for *K* = 2–3. (c) Principal component analysis of the gene-expression profiles of the two m5C isoforms: the transcriptomes of different modification patterns are significantly different. (d) Volcano plot showing the difference in the two m5C isoforms. (e) Heatmap showing the difference in the two m5C isoforms.

To investigate the modification pattern of m5C in SCI, we undertook unsupervised consensus clustering analysis of SCI samples based on expression of 16 m5C-based regulators of RNA modification (Figures 7(a) and 7(b)). Clustering had better stability if *K* = 2. PCA revealed that two molecular subtypes of m5C had differences (Figure 7(c)). Then, we analyzed the differences in gene expression between these two molecular subtypes of m5C using heatmaps. The volcano plot (Figure 7(d)) and heatmap (Figure 7(e)) represented differentially expressed genes between these two molecular subtypes.

### 3.6. Functional-Enrichment Analysis of Differentially Expressed Genes with Different Patterns of Basing on the Expression of 16 m5C Regulators

**Figure 8:**
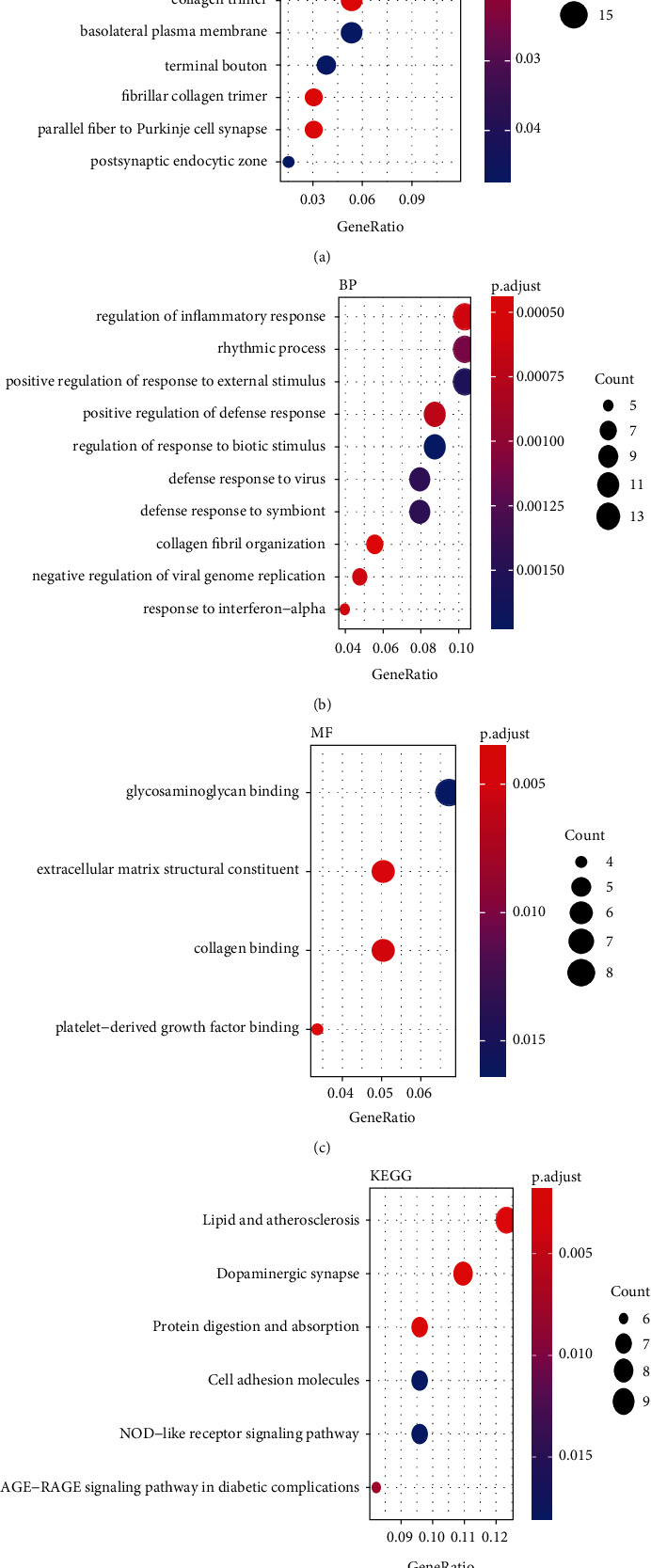
Analysis of functional enrichment (using the Gene Ontology database) and signaling pathways (using the Kyoto Encyclopedia of Genes and Genomes database). (a) Analysis of functional enrichment. The abscissa is the gene ratio, and the ordinate is the name of the signaling pathway. The node size indicates the number of genes enriched in the signaling pathway. The node color indicates the *p*-value. The top-10 cellular component is shown. (b) Top-10 biological process. (c) Molecular function. (d) Analysis of signaling-pathway enrichment. The abscissa is the gene ratio, and the ordinate is the name. The node size indicates the number of genes enriched in the signaling pathway. The node color indicates the *p*-value.

We wished to determine the relationship between differentially expressed genes among different patterns of the expression of 16 m5C regulators and BP, MF, and CC signaling pathways. Hence, we undertook functional-enrichment analysis using the GO database (Figure 8). For BP, the primary enrichment was in “regulation of inflammatory response,” “positive regulation of response to external stimulus,” and “positive regulation of defense response.” For CC, the primary enrichment was in “collagen-containing extracellular matrix,” “extracellular matrix,” and “external encapsulating structure.” For MF, the primary enrichment was in “platelet-derived growth factor binding,” “extracellular matrix structural constituent,” and “collagen binding” (Figures 8(a), 8(b), and 8(C); Table 2). Analysis of signaling-pathway enrichment using the KEGG database revealed that the primary enrichment was mainly in “lipid and atherosclerosis,” “dopaminergic synapse,” and “protein digestion and absorption” (Figure 8(d); Table 3).

### 3.7. PPI Analysis and Screening of Hub Genes

**Figure 9:**
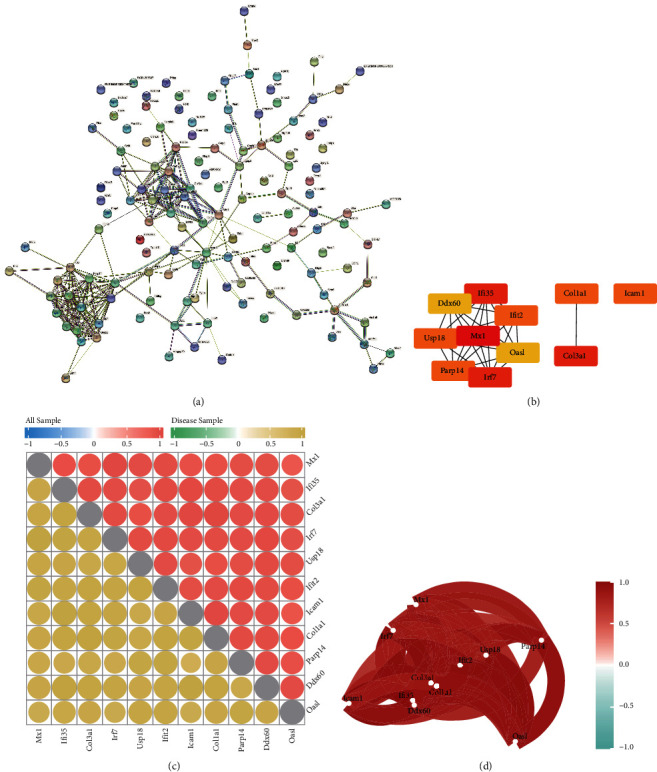
Protein–protein interaction (PPI) network and screening for hub genes. (a) PPI network of genes with differential 5-methylcytosine-based modification patterns in the Search Tool for the Retrieval of Interacting Genes/Proteins database. (b) Screening of 11 hub genes. (c) Heatmap showing correlation of hub genes. The upper-right half is the correlation in all samples. The lower-left half is the correlation in a spinal cord injury sample. The size and color of the nodes represent the size of the correlation. (d) Network diagram showing the correlation in expression of hub genes.

**Figure 10:**
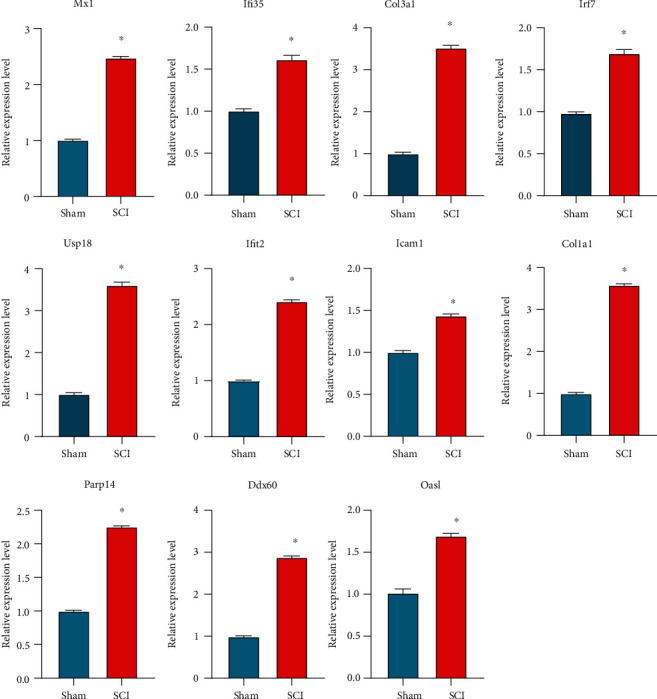
Validation of the hub genes involved in 5-methylcytosine-based modification of RNA by reverse transcription-quantitative polymerase chain reaction (RT-qPCR). RT-qPCR verification of 11 hub genes in samples from rats suffering from spinal cord injury and samples from healthy rats. Data are the mean ± SE. ∗*p* < 0.05.

We constructed a PPI network associated with differentially expressed genes between different patterns of m5C modification (Figure 9(a)). After that, we imported the relationship of protein interactions into Cytoscape and used the cytoHubba plugin to screen for hub genes. We screened 11 hub genes: myxovirus resistance 1 (*Mx1*), interferon inducible protein 35 (*Ifi35*), collagen type III alpha 1 chain (*Col3a1*), interferon regulatory factor 7 (*Irf7*), *Usp18*, interferon-induced protein with tetratricopeptide repeat 2 (*Ifit2*), intercellular adhesion molecule-1 (*Icam1*), collagen type I alpha1 (*Col1a1*), poly (ADP-ribose) polymerase family, member 14 (*Parp14*), DEXD/H box helicase 60 (*Ddx60*), and oligoadenylate synthetase-like (*Oasl*; Figure 9(b)). We explored the internal correlation between these 11 hub genes. The heatmap and network map showed that all hub genes had strong correlations (Figures 9(c) and 9(d)). Finally, we quantified the expression of these hub genes in rat samples using reverse transcription-quantitative PCR (RT-qPCR). The SCI group had considerably higher expression of these 11 hub genes than the sham group (Figure 10).

### 3.8. The Biological Function of Hub Genes

**Figure 11:**
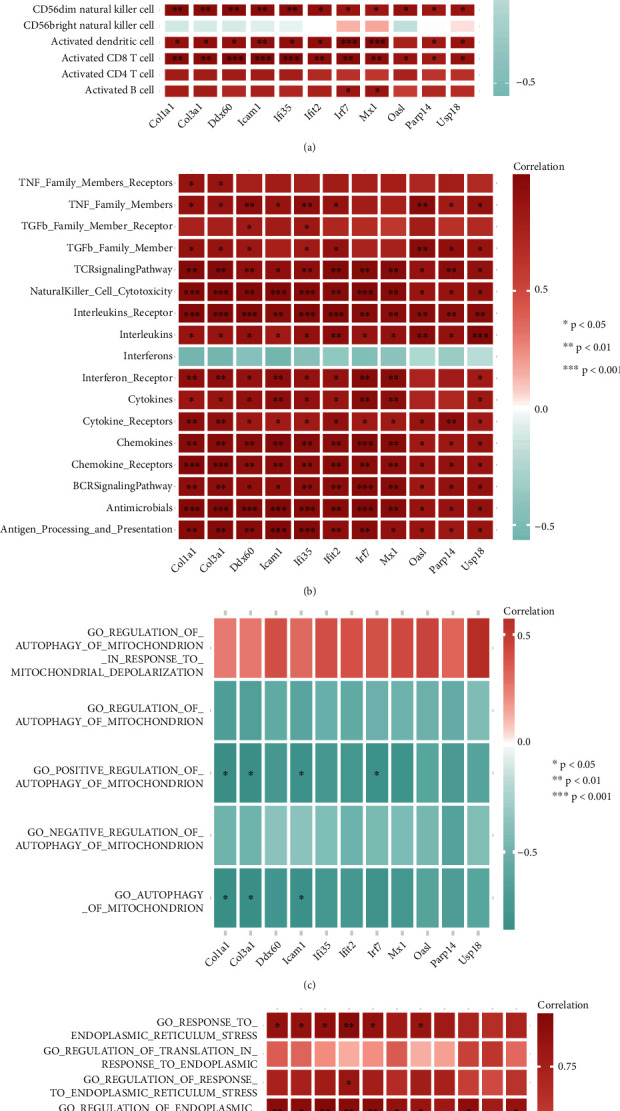
Correlation of expression of hub genes with immunity, endoplasmic reticulum stress (ERS), and mitophagy-related pathways. (a) Overall heatmap of the correlation between hub-gene expression and abundance of immune cells. (b) Overall heatmap of the correlation between hub-gene expression and immune processes. (c) Overall heatmap of the correlation between hub-gene expression and mitophagy-related pathways. (d) Global heatmap of correlations between hub-gene expression and ERS-related pathways.

We explored whether these 11 hub genes could regulate the immune process and immune microenvironment by analyzing the correlation between hub genes and immune cells and immune processes (Figures 11(a) and 11(b)). Expression of these 11 hub genes was positively correlated with immune cells and immune processes. Studies have shown that ERS and mitophagy have essential roles in SCI [29, 30]. Hence, we used GSVA to calculate the related signaling pathways' scores and the correlation of 11 hub genes with these signaling pathways (Figures 11(c) and 11(d)). Expression of these hub genes did not have a close correlation with mitophagy but was positively correlated with ERS, suggesting that there may be differences in ERS between different m5C subtypes.

## 4. Discussion

SCI refers to severe damage to the central nervous system that results in loss of motor and sensory functions. Unrepairable nerve injury has been considered to be the essential reason causing treatment failure for SCI [8]. Therefore, discovering the regulatory features that affect neural repair and then guiding SCI treatment are rational approaches.

Many recent studies have reported that m5C-based regulators have essential roles in various human diseases [32]. Although the importance of m5C-based modification in SCI has been emphasized, research on m5C-based modulators in SCI is in its infancy. Thus, we performed the systematic bioinformatics analysis of m5C-based regulators in SCI. This study would provide reliable guidelines for future specific experimental investigations of SCI as well as novel opportunities for the development of effective therapies.

We described the expression profiles of 16 m5C genes between SCI and sham groups. These results from the publicly available GEO database showed that one writer (Dnmt1) and five readers (Mbd2, Uhrf1, Ung, Zbtb38, and Zbtb4) had the significant expression, which suggested their possible functional importance in SCI. Similarly, the modification of m5C is also catalyzed by methyltransferases. Dnmt1 deletion causes severe disruption in the epidermal structure and homeostasis, thereby triggering a massive innate immune response and infiltration of immune cells [33]. As reported by Wang et al., Mbd2 expression is reduced in the lumbar spinal cord in rats 14 days after surgery [34]. The deletion of Mbd2 rendered it incapable of “reading” methylation information, which disrupted the homeostasis of the T-bet/H 2.0-like homeobox axis and inhibited the differentiation of T-helper type 17 cells, thereby protecting against experimental autoimmune encephalomyelitis [35]. The primary and most effective uracil DNA glycosylase (Udg) for removal of uracil from nuclear DNA is uracil DNA glycosylase 2 (hUNG2), whereas human uracil DNA glycosylase 1 (hUNG1) is a mitochondrial splice variant. hUNG is highly effective at removing deaminated cytosine and mis-incorporated uracil from single-stranded DNA, which is abundant in replicating cells [36]. High expression of Zbtb38 can promote autophagy and partly rescue the secondary damage caused by SCI [37]. Zbtb4 is a mammalian DNA-binding protein, contains C_2_H_2_ zinc fingers and a BTB/POZ domain, and functions as a transcriptional repressor [38]. Zbtb4 is responsible for p53-induced apoptosis and cell-cycle arrest because it regulates the expression of cyclin-dependent kinase inhibitor-1A [39].

The RF method was a learning method based on a decision tree. It identified the characteristic sets with random selection and ramdom sampling. Additionally, It was also difficult to develop overfitting results and had an adequate anti-noise capability [40]. In recent years, the RF algorithm has become increasingly popular for data prediction. LASSO regression is also a comprehensive machine-learning technique for selecting variables by identifying those with the lowest classification error probability. We analyzed the discriminatory power of m5C-related genes in a SCI group *versus* a normal group using the RF approach. Results showed that the model scores of the SCI group and normal group were significantly different, m5C-related genes had good predictive ability, and the difference in risk between the SCI group and normal group was significant.

To further investigate the differential gene expression of m5C in rats suffering from SCI and normal rats, we continued to examine the correlation of m5C-based modulators with the immune microenvironment. Interestingly, Mbd2 and Ung may positively regulate the immune microenvironment, and Zbtb4 may negatively regulate the immune environment in SCI tissues. Mbd2 has been shown to suppress inflammation and disease through regulation of T-cell recruitment and crosstalk between innate epithelial cells [41]. UNG deficiency has been shown to inhibit expansion of B cell clones in the germinal centers of mice and inhibit expression of tumorous B cells [42]. Zbtb4 promotes transcription activation for Yip1 domain family 3 (YIPF3) expression, so YIPF3 enhances the response to immune stimulation [43]. To further explore the pattern of m5C-based modification in SCI, we undertook unsupervised consensus clustering analysis. Results showed that if *K* = 2, clustering had better stability. PCA showed that the two molecular subtypes of m5C were well differentiated. Although we revealed the differential expression of m5C-based genes in rats suffering from SCI and normal rats, the complex mechanisms of m5C and SCI must be investigated in the future.

To further explore the downstream MFs and mechanisms involved in SCI-related m5C regulators, we undertook the analysis of functional enrichment based on the genes related to the two molecular subtypes of m5C. Some of these functions and signaling pathways have been reported to exacerbate the onset and development of SCI [44, 45]. Next, we screened 11 hub genes and carried out correlation analysis: expression of all hub genes was strongly correlated. We also verified expression of these hub genes between the SCI group and normal group using RT-qPCR: expression of these hub genes was higher in the SCI group than that in the sham group. Moreover, expression of these 11 hub genes was positively associated with the abundance of immune cells and immune processes. Simultaneously, GSVA was used to calculate the correlation of expression of these 11 hub genes with signaling pathways. Expression of these hub genes was not highly correlated with mitophagy but was positively correlated with ERS, which suggested that ERS may differ between different patterns of m5C-based RNA modification.

Our study had two main limitations. First, the primary published data were from retrospective studies, so we could not download additional information on statistical analysis and clinical characteristics of SCI (e.g., severity and complications). Second, due to the limitations of bioinformatics analysis, further studies must be carried out to investigate the repairing effect of m5C-based RNA methylation in SCI.

## 5. Conclusions

We demonstrated the expression and potential functions of regulators of m5C-based RNA methylation in SCI using bioinformatics analysis. We documented higher expression of Mbd2, Uhrf1, Ung, Zbtb38, and Zbtb4 in samples from rats suffering from SCI than that in samples from healthy rats, and Zbtb4 expression in rats suffering from SCI was lower than that in the control group. Zbtb4 may negatively regulate the immune environment, and Zbtb4 may be a promising therapeutic target for SCI. We documented significant associations in expression with some key genes (*Mx1*, *Ifi35*, *Col3a1*, *Irf7*, *Usp18*, *Ifit2*, *Icam1*, *Col1a1*, *Parp14*, *Ddx60*, and *Oasl*) based on these regulators of m5C-based RNA methylation. Expression of these gene sets was positively correlated with ERS, and may promote the development and progression of SCI.

## Figures and Tables

**Table 1:** Messenger RNA-specific primers of hub genes.

Gene	Primer	Sequence (5′—3′)	Polymerase chain reaction products (bp)
*Mx1*	Forward	CTAAGCAGGAGACTGTGCCT	86
	Reverse	CAGGTGGATTCGGTTGGACA	
*Ifi35*	Forward	CCAAAGTGGTTGAGCGGTTG	158
	Reverse	AAAGCCACTGACTAGCACCC	
*Col3a1*	Forward	AGGGCAGGGAACAACTGATG	115
	Reverse	GGTCCCACATTGCACAAAGC	
*Irf7*	Forward	GAAGTGAGGCCTGGGCTG	142
	Reverse	AAGATAAAGCGCCCTGTGCT	
*Usp18*	Forward	TGAGAGGGTCCTTTCTGGCT	189
	Reverse	CTTCCTCCAGAGCTGAGTGC	
*Ifit2*	Forward	GGCCATTGCAAACTACCGTC	93
	Reverse	GTGTTGTCAGGAGACAGGCT	
*Icam1*	Forward	TTTCAGCTCCCATCCTGACC	144
	Reverse	GGGAAGTACCCTGTGAGGTG	
*Col1a1*	Forward	GGAGAGAGCATGACCGATGG	184
	Reverse	GGGACTTCTTGAGGTTGCCA	
*Parp14*	Forward	GTGCAGGCTGTGAAGAGAGT	141
	Reverse	GCCAAGGAAGGAGGCAATCT	
*Ddx60*	Forward	TGACTCCGAAGCTTCCGCTA	188
	Reverse	CAGCATGCGCGTTAAGACAG	
*Oasl*	Forward	TACTGCCAGACGCTCTTGTG	180
	Reverse	CAACCTGAAGCAGGGTCCAT	
*β*-*Tubulin*	Forward	TAACGAGGCCCTCTACGACA	77
	Reverse	CGAGATGGTTCAGGTCTCCG	

**Table 2:** Analysis of functional enrichment of differentially expressed genes between different 5-methylcytosine subtypes using the Gene Ontology (GO) database.

Ontology	ID	Description	*p*-value adjusted
BP	GO:0030199	Collagen fibril organization	0.000444603
BP	GO:0050727	Regulation of inflammatory response	0.000617651
BP	GO:0045071	Negative regulation of viral genome replication	0.000617651
BP	GO:0035455	Response to interferon-alpha	0.000617651
BP	GO:0031349	Positive regulation of defense response	0.000710318
BP	GO:0048511	Rhythmic process	0.00111207
BP	GO:0051607	Defense response to virus	0.001422501
BP	GO:0140546	Defense response to symbiont	0.001422501
BP	GO:0032103	Positive regulation of response to external stimulus	0.00154312
BP	GO:0030199	Collagen fibril organization	0.000444603
CC	GO:0062023	Collagen-containing extracellular matrix	1.55 × 10^−5^
CC	GO:0031012	Extracellular matrix	1.55 × 10^−5^
CC	GO:0030312	External encapsulating structure	1.55 × 10^−5^
CC	GO:0005581	Collagen trimer	1.55 × 10^−5^
CC	GO:0005583	Fibrillar collagen trimer	3.52 × 10^−5^
CC	GO:0098688	Parallel fiber to Purkinje cell synapse	0.002892418
CC	GO:0009897	External side of plasma membrane	0.006331384
CC	GO:0098843	Postsynaptic endocytic zone	0.047250858
CC	GO:0043195	Terminal bouton	0.047250858
CC	GO:0016323	Basolateral plasma membrane	0.047250858
MF	GO:0048407	Platelet-derived growth factor binding	0.000294167
MF	GO:0005201	Extracellular matrix structural constituent	0.00184744
MF	GO:0005518	Collagen binding	0.001997328
MF	GO:0005539	Glycosaminoglycan binding	0.015404978

**Table 3:** Analysis of signaling-pathway enrichment of differentially expressed genes between different 5-methylcytosine subtypes using the Kyoto Encyclopedia of Genes and Genomes database.

ID	Description	*p*-value adjusted
rno04728	Dopaminergic synapse	0.001811519
rno04974	Protein digestion and absorption	0.001811519
rno05417	Lipid and atherosclerosis	0.002952352
rno04933	AGE–RAGE signaling pathway in diabetic complications	0.007778799
rno04514	Cell adhesion molecules	0.01804195
rno04621	NOD-like receptor signaling pathway	0.01804195
rno05031	Amphetamine addiction	0.054241866
rno05164	Influenza A	0.054241866
rno04670	Leukocyte transendothelial migration	0.054241866
rno05416	Viral myocarditis	0.08018878

## Data Availability

The Gene Expression Omnibus database (https://www.ncbi.nlm.nih.gov/geo/) provides the microarray data to support the findings of this study.
